# A Monoclonal–Monoclonal Antibody Based Capture ELISA for Abrin

**DOI:** 10.3390/toxins9100328

**Published:** 2017-10-18

**Authors:** Christina C. Tam, Luisa W. Cheng, Xiaohua He, Paul Merrill, David Hodge, Larry H. Stanker

**Affiliations:** 1 Foodborne Toxin Detection and Prevention Research Unit, Western Regional Research Center, Agricultural Research Services, United States Department of Agriculture, 800 Buchanan Street, Albany, CA 94710, USA; christina.tam@ars.usda.gov (C.C.T.); luisa.cheng@ars.usda.gov (L.W.C.); xiaohua.he@ars.usda.gov (X.H.); paul.merrill@ars.usda.gov (P.M.); 2 United States Department of Homeland Security, Washington, DC 20528, USA; david.hodge@hq.dhs.gov

**Keywords:** abrin, *abrus precatorius*, ELISA, monoclonal antibodies, ribosome-inactivating protein

## Abstract

Abrin, one of the most highly potent toxins in the world, is derived from the plant, *Abrus precatorius*. Because of its high toxicity, it poses potential bioterror risks. Therefore, a need exists for new reagents and technologies that would be able to rapidly detect abrin contamination as well as lead to new therapeutics. We report here a group of abrin-specific monoclonal antibodies (mAbs) that recognize abrin A-chain, intact A–B chain toxin, and agglutinin by Western blot. Additionally, these mAbs were evaluated for their ability to serve as capture antibodies for a sandwich (capture) ELISA. All possible capture–detector pairs were evaluated and the best antibody pair identified and optimized for a capture ELISA. The capture ELISA based on this capture–detector mAb pair had a limit of detection (L.O.D) of ≈1 ng/mL measured using three independent experiments. The assay did not reveal any false positives with extracts containing other potential ribosome-inactivating proteins (RIPs). Thus, this new capture ELISA uses mAbs for both capture and detection; has no cross-reactivity against other plant RIPs; and has a sensitivity comparable to other reported capture ELISAs using polyclonal antibodies as either capture or detector.

## 1. Introduction

Abrin and ricin are both members of the Type II family of ribosome-inactivating proteins (RIP) that inhibit eukaryotic protein synthesis leading to apoptosis and cell death. Abrin is found in jequirity seeds or rosary peas of the plant, *Abrus precatorius* L*.*, and native to tropical/subtropical regions of the world. Abrin like ricin is considered a Select Agent due to its potential use in bio warfare. Abrin is a heterodimeric protein toxin consisting of an A-chain and a B-chain linked together with a single disulfide. The catalytic active A-chain is an *N*-glycosidase that deadenylates the rat 28S ribosomal RNA at position 4324 [1] thereby preventing ribosomes from binding to elongation factor (EF) 1 and 2 leading to inhibition of protein synthesis and eventual cell death [2,3,4,5,6,7]. Abrin B-chain is a lectin that binds to cell surface carbohydrate receptors facilitating receptor-mediated endocytosis of the A–B toxin. 

Abrin isolated from *Abrus precatorius* seeds is a heterogeneous mixture [7,8,9]. Multiple isoforms have been reported, and each form has different toxicity [8,9,10,11,12,13,14]. In addition to these toxin isoforms, the presence of a 120 kDa heterotetrameric species, referred to as the agglutinin (APA-1), also can be isolated from seed preparations. APA-1 consists of two A-chains and two B-chains that are stabilized through hydrophilic and hydrophobic interactions [15]. Though APA-1 and abrin toxin isoforms have very similar sequences, APA-1 has reduced toxicity as compared to abrin [8,16,17].

Abrin intoxication can occur via multiple routes that affect its toxicity. The routes of intoxication are gastrointestinal, inhalational, or cutaneous. Abrin intoxication cases have been attributed to the accidental ingestion of *A. precatorius* seeds by children. Since there is no antidote for abrin poisoning, the only available treatment is supportive care to mitigate the effects of the toxin. In the case of abrin intoxication via the ingestion, the current treatment consists of inducing emesis, gastric lavage, activated charcoal, and whole bowel irrigation [3]. The LD_50_ of abrin for humans has been reported to range from 10–1000 μg/kg via oral ingestion and 3.3 μg/kg if injected [18]. In mice, abrin has been shown to be 31.4 times more lethal than ricin with an LD of 0.7 μg/kg vs. 22 μg/kg when given intravenously [19]. 

Multiple technologies (electrospray MS, portable sensor, etc.) have been developed to detect l-abrine (*N*-methyltryptophan) [18,20,21] as a biomarker for abrin exposure. ELISA, ECL, and lateral flow technologies have been reported to detect abrin. Earlier work reported that polyclonal–monoclonal antibody, as well as polyclonal–polyclonal based capture ELISAs and ECL assays have limits of detection (LODs) ranging from 0.1–0.5 ng/mL in buffer [15]. In the same study, the authors report that the ECL platform had LODs from 0.1–0.5 ng/mL in different food matrices whereas the ELISAs had higher LODs (0.5–10 ng/mL). Other studies detecting abrin in food using monoclonal antibodies as capture with polyclonal antibodies as detector reported an LOD of ≈0.7 ng/mL in PBS or PBSTM [22]. A monoclonal capture with a polyclonal detector ELISA was reported to have a LOD in buffer of ≈0.5 μg/L (0.5 ng/mL) [23]. We speculated that a capture ELISA based on monoclonal antibodies for both capture and detection could be developed to have sensitivities comparable to those seen in literature. Monoclonal antibodies have defined epitopes and they represent a more consistent source for a critical reagent in comparison to polyclonal antibodies. Furthermore, they could potentially be used as therapeutics to neutralize toxins in the future.

To help mitigate a potential bioterrorist event using abrin, new reagents and technologies that would rapidly detect this toxin are highly desirable. In this study, we characterized a group of monoclonal antibodies that led to the development of a monoclonal-monoclonal based capture ELISA for abrin detection with a LOD comparable to those reported in literature and commercially available.

## 2. Results

### 2.1. Characterization of Anti-Abrin Monoclonal Antibodies

Anti-abrin mAbs from ten hybridoma cell lines were grown as previously described [24] and purified from the ascites fluid by affinity chromatography on Protein-G Sepharose. The antibodies referred to as LS02ABx, LS03ABx, LS04ABx, LS05ABx, LS06ABx, LS07ABx, LS08ABx, LS10ABx, LS11ABx, and LS13ABx. Seven of the mAbs have IgG1 kappa heavy chains with kappa light chains, while the rest were either IgG2a/IgG2b kappa and one had a lambda light chain (Table 1). Each of the antibodies was successfully labeled with biotin, with an average of 2–4 biotin molecules per IgG molecule (Table 1).

### 2.2. Silver Stain and Western Blots

**Figure 1:**
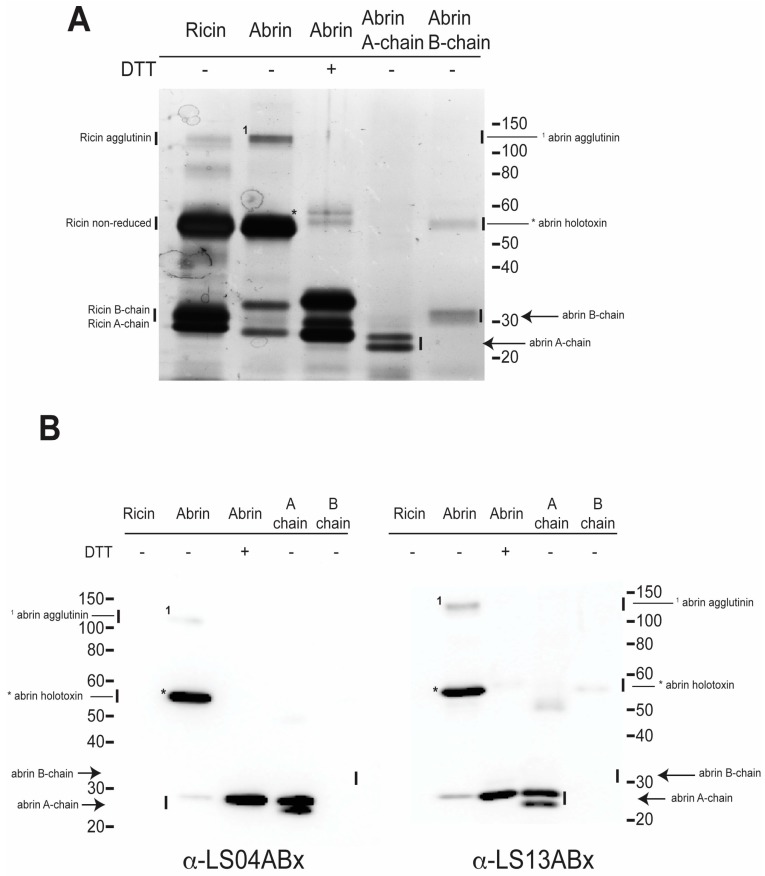
SDS-PAGE analysis of toxin complexes and subunits. 500 ng per lane of sample (ricin, abrin, abrin A-chain, abrin B-chain) treated or not treated with 0.05 M DTT was loaded onto a NuPAGE 4–12% Bis-Tris gel and subjected to SDS-PAGE electrophoresis. Gels were processed for silver staining or Western blotting. (**A**) Silver staining with the SilverXpress. (**B**) Representative Western blots probed with mAbs LS04ABx and LS13ABx. Similar exposure times and images were obtained for the remaining mAbs (data not shown). *—Abrin holotoxin non-reduced; ^1^—agglutinin, and corresponding arrows denote A- and B-chains.

In an effort to determine if these mAbs bound the A- or B-chain of the toxin each mAb was used to probe Western blots. Electrophoresis of our abrin and ricin stocks followed by silver staining revealed multiple bands (Figure 1A). Ricin consists of the holotoxin, the agglutinin, and the individual A and B subunits when electrophoresed without DTT. Similarly, non-reduced abrin consists primarily of the holotoxin (*), some agglutinin (^1^), A-chain, and B-chain. Addition of DTT to the abrin material resulted in a significant reduction of the agglutinin and the holotoxin but an increase of the A and B subunits. The abrin A-chain control sample (without reducing agent) has two predominant A-chain isomers. The non-reduced abrin B-chain control shows the expected B-chain as well as a higher molecular weight species that is most likely the holotoxin. The same samples were subjected to immunoblotting with each of the 10 LSABx monoclonal antibodies to determine if there was cross-reactivity with ricin as well as to determine which abrin chain, A or B, the mAbs bound. Typical results are shown in Figure 1B for mAbs LS04ABx and LS13ABx but similar results were obtained for each of the mAbs (data not shown). All were observed to bind strongly to abrin holotoxin and A-chain. Binding to the agglutinin is weak but present on the immunoblots. None of the mAbs recognize the abrin B-chain or any form of ricin. 

### 2.3. Monoclonal Antibody Binding to Abrin Toxin and Individual A and B-Chain Subunits

**Figure 2:**
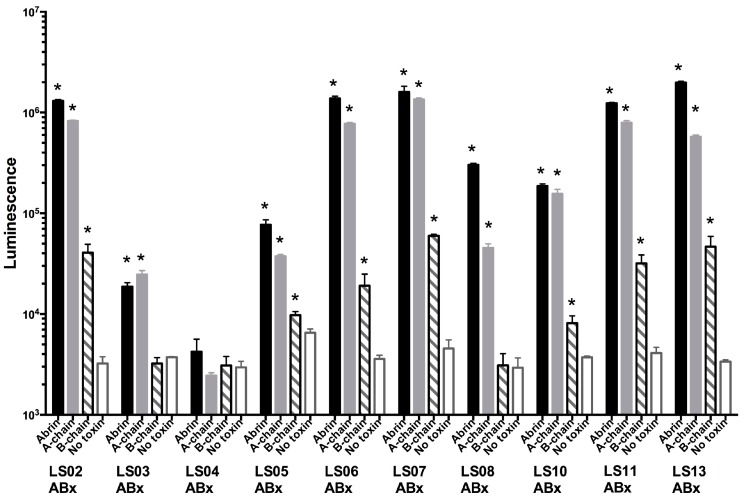
LSABx monoclonal antibodies recognize the abrin A-chain in a direct ELISA. Samples of 1 μg/mL of abrin, A-chain, B-chain, or no toxin were assayed for each of the 10 LSABx-biotinylated antibodies at a concentration of 1 μg/mL. Luminescence was measured from triplicate wells; error bars equal ± one standard deviation (SD). Statistical significance was determined by two-tailed unpaired Student’s *t*-test comparing each column with the no toxin control for each set of mAbs, (*) *p*-values < 0.05 are considered significant. Abrin toxin (black columns); A-chain (grey columns); B-chain (hashed columns); and no toxin (open grey columns).

The Western blots indicated that these antibodies were directed against the A-chain, we used a direct ELISA to confirm this specificity. Abrin toxin, A-chain, and B-chain were used to coat 96-well plates and binding of biotin-labeled anti-abrin mAbs was measured. The results of these experiments are summarized in Figure 2. With the exception of mAb LS04ABx, which bound poorly to abrin immobilized on plastic ELISA plates, the Abs strongly bound abrin toxin (black columns) and abrin A-chain (grey columns) compared to no toxin control (open grey columns), *p*-values < 0.05. In contrast, greatly reduced or no binding to B-chain (hashed columns) was observed as compared to the abrin toxin or abrin-A-chain. The weak binding (* *p* < 0.05) observed in ELISA by some of the mAbs to the B-chain standard may reflect the observation in the above experiments (Figure 1A) suggesting some A-chain contamination of the B-chain preparation. B-chain binding is at least an order of magnitude less than the signal seen with either abrin or abrin A-chain after the signal of the control is subtracted (Figure 2). 

### 2.4. Monoclonal–Monoclonal Capture ELISA for Abrin

The 10 anti-Abrin mAbs were evaluated for their ability to function as either capture or detector antibodies in a capture (sandwich) ELISA format. These results expressed as the lower limit of detection (LOD) for each possible antibody combination are summarized in Table 2. They are expressed as the value in ng/mL that gave a signal above the average signal of the no-toxin control plus three SD. Clearly the majority of antibodies failed to bind toxin in solution, to capture the toxin. Of the 100 possible combinations only 4 capture–detector pairs resulted in LODs ≤10 ng/mL. These capture-detection pairs were LS04ABx-LS11ABx, LS04ABx-LS13ABx, LS06ABx-LS08ABx, and LS08ABx-LS11ABx. Since these represent preliminary experiments not using the most sensitive substrate, they were further evaluated.

**Figure 3:**
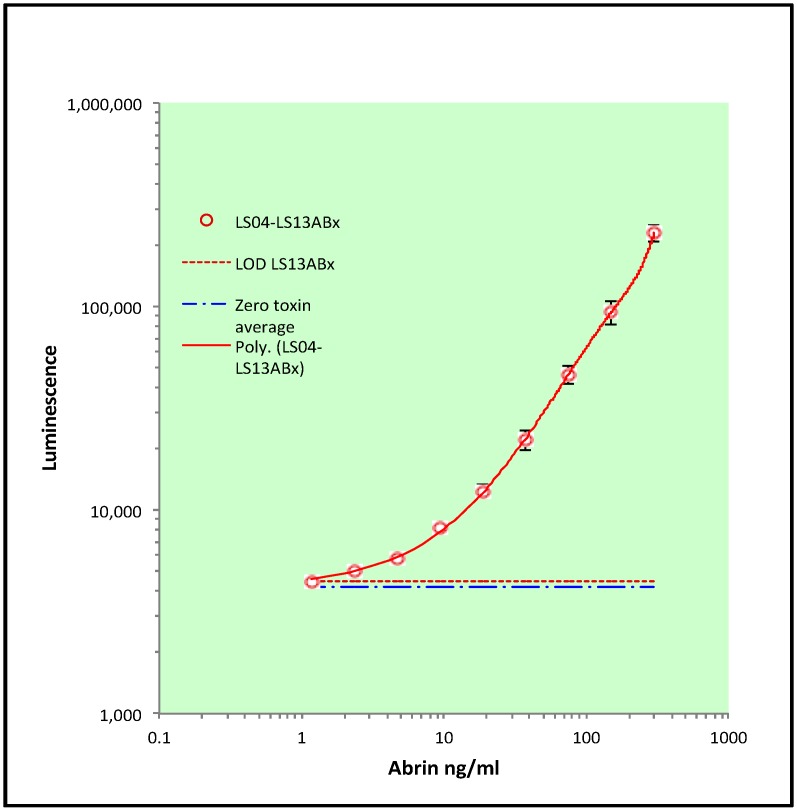
Capture ELISA optimized for detection of Abrin. Mab LS04ABx used as the capture reagent at 2 µg/mL. Detector antibody LS13ABx was used at 1 µg/mL. Representative capture ELISA result showing one out of three independent experiments (see Figure S1 for the remaining experiments). Points represent the average of a triplicate (*n* = 3), error bars = standard deviation. Solid lines show 4-Parameter curve fitting. Red dashed line represents the lowest detection level cut offs calculated as the average of the zero plus three standard deviations. Blue broken line represents the average value of the zero toxin control. Statistical significance was determined by two-tailed unpaired Student’s *t*-test, *p* < 0.05 considered statistically significant for all points compared to the average zero toxin value.

These four capture–detector mAb pairs were further optimized by adjusting antibody concentrations and use of SuperSignal Femto Max Sensitivity substrate (data not shown). Results using mAb LS04ABx as a capture with biotin-labeled mAb LS13ABx as a detector are shown in a representative ELISA curve in Figure 3 representing one independent experiment consisting of three replicates. The assay has a good dynamic range with starting concentrations of 300 ng/mL with sequential two-fold dilutions of the toxin in buffer. Each point on the curve was statistically significant compared with the no toxin control. The limit of detection was determined by calculating the average of the zero toxin controls plus 3 times the standard deviation. In Figure 3, the LOD for this assay was 1.1 ng/mL. An additional two independent experiments each with replicates (*n* = 6) was performed on different days with different buffers and toxin stocks (Supplemental Figure S1). The LOD’s from these experiments were ≈1 ng/mL and 0.8 ng/mL. The overall LOD calculated from the three independent experiments was ≈1 ng/mL ± 0.15.

### 2.5. Specificity of Abrin Capture ELISA against Other Plant Ribosome-Inactivating Proteins (RIPs)

Ribosome-inactivating proteins from both Type I and Type II families are prevalent in the plant kingdom. One must take into account whether there may be cross-reactivity between abrin with these other RIPs resulting in ‘false’ positives in an assay. Earlier studies had tested the specificity of a commercially available lateral flow device against 35 near neighbor crude extracts [25]. The authors reported that the extracts with the exception of *Abrus laevigatus* tested negative at a concentration of 10 μg/mL. Abrin and the identical extracts used above in [24] plus an additional 10 extracts were tested here. These extracts were tested in triplicate at 3 μg/mL with the optimized capture ELISA shown in Figure 3. All 46 near neighbor extracts tested here were negative (Table 3) including *Abrus laevigatus*. Only abrin from the *Abrus precatorius* extract was positive in the assay.

### 2.6. MAb Neutralization of Abrin Cytotoxicity in Cells

Selected mAbs were premixed with toxin in order to determine if they could neutralize the toxin. In these experiments abrin was used at 5 ng/mL and mixtures of toxin and antibody contained the mAb at 50 µg/mL. These data, are summarized in Table 4, and demonstrate that administration of the antibody alone had no toxic effect on the Vero cell growth. Further, premixing toxin with mAbs LS02ABx, LS03ABx, LS04ABx, LS07ABx, LS08ABx, LS10ABx, or LS11ABx had only minimal effects on abrin cytotoxicity. In contrast, mixing mAb LS13ABx with toxin showed a relative toxicity of 54%. Mixing three mAbs, LS02 + 07 + 13ABx resulted in an even greater decrease in relative cytotoxicity, to 41% as compared with exposure to abrin by itself. As a control for antibody specificity, ricin cytotoxicity was observed not to be inhibited by the same antibodies used in the Vero cell assay (Table S1). 

## 3. Discussion

Due to the highly toxic nature of abrin resulting in its classification as a select agent we evaluated a set of mAbs to abrin. Both polyclonal- and polyclonal/monoclonal-based capture immunoassays have been described [15,22,23,26]. In addition, a sensitive lateral flow immunoassay (LFA) incorporating up-converting phosphor technology (UPT) and laser interrogation has recently been described [27]. The authors developed the LFA with up converting phosphor-conjugated antibodies that would required a specially built reader that excites the lanthanide-doped crystals by infrared light and emits visible light. The abrin-UPT-LFA had detection limits as low as 0.1 ng/mL. A monoclonal capture ELISA with an LOD of 7.8 ng/mL [27] and use of aptamers for abrin detection in a colorimetric assay with an LOD of 0.05 nM (3.1 ng/mL) [28] also have been reported. Immunoassay performance fundamentally depends on the performance of the antibodies used as well the assay format. Thus it is important to have a large selection of well-characterized antibodies available for assay development. However, it is important that monoclonal-based capture immunoassays have similar LOD’s as compared to those reported in literature. Previous works on detection technologies for abrin have been dependent on polyclonal and monoclonal antibodies for both capture and detector antibodies [15,22,23]. Monoclonal-monoclonal based ELISA would not be subjected to the variability inherent in assays based on polyclonal antisera, the assay should be more consistent over time and readily transported to different capture immunoassay formats. 

We report here the results of our analysis of ten anti-Abrin mAbs. All demonstrated specific binding to abrin A-chain and to holotoxin on Western blots. This was further validated by our observations that the antibodies bound the A-chain but not the B-chain in ELISA using purified A- and B-chain. Since the antibodies specifically bound the A-chain and the holotoxin, the binding epitope is not obscured in the A–B holotoxin. Antibody binding to the agglutinin also was observed. Additionally, there was no cross-reactivity with any form of ricin (Figure 1). The best capture mAb was LS04ABx, which poorly bound plate-immobilized abrin by ELISA. These observations suggest that LS04ABx recognizes a complex or conformational epitope that is masked or modified when toxin is absorbed onto a plastic surface. We have observed this before in our studies to generate capture antibodies to botulinum neurotoxin [29]. Perhaps this is a result of both toxins being A-B toxins.

Capture–detector pairs were evaluated for their ability to perform in a capture ELISA (Table 2). Of all the possible pair combinations, 4 capture–detector pairs (LS04ABx-LS11ABx; LS04ABx-LS13ABx; LS06ABx-LS08ABx; and LS08ABx-LS11ABx) were found to have LODs low enough to be useful. These assays were further optimized with the aim of reducing their LODs (≤10 ng/mL). One capture–detector pair with LS04ABx as capture and LS13ABx as detector had a LOD ≈ 1 ng/mL (Figure 3). When this capture ELISA was evaluated for potential cross-reactivity against other plant RIPS, no false positives were observed (Table 3). The cytotoxicity studies, while preliminary, suggest that mAbLS13ABx can neutralize abrin toxin specifically but that mixing multiple antibodies improved neutralization (Table 4, Table S1). These results are supported by other studies that demonstrated mixtures of anti-botulism mAbs were more effective than single mAbs [30,31,32,33].

Therefore, we report the development of a new monoclonal-monoclonal based capture ELISA that has high sensitivity with a LOD ≈ 1 ng/mL ±0.15. This capture ELISA detects the holotoxin as well as the A-chain. The lack of false positives when 46 abrin/ricin near neighbor RIPs suggests that the assay is highly specific for abrin. These new monoclonal antibodies could be developed further in other detection assays using different technologies to increase the sensitivity of these reagents i.e., up-converting phosphor technology or used in conjunction with other abrin monoclonal/polyclonal antibodies. Furthermore, the neutralization potential of these mAbs should be tested in vivo with the mouse bioassay to validate whether or not these are truly neutralization antibodies and if so, determine the temporal effectiveness of the antibody-based protection towards abrin intoxication.

## 4. Materials and Methods

### 4.1. Reagents

Pure ricin (RCA-II, Cat. #L1090) was obtained from Vector Laboratories (Burlingame, CA, USA) as a 5 mg/mL solution and stored at 4 °C. Abrin toxin (mixed isomers, Cat# ABR-1) was purchased from Toxin Technologies, Inc. (Sarasota, FL, USA) as a lyophilized material at 1 mg/vial. The contents of the vial were dissolved by addition of 1 mL of 1x phosphate buffered saline (PBS) pH 7.4 to give a 1 mg/mL stock stored at 4 °C. Abrin A-chain (BEI Resources, NR-43945) and Abrin B-chain (BEI Resources, NR-43946) were supplied as 1 mg/mL stocks stored at −20 °C. Hybridoma cell lines secreting anti-abrin monoclonal antibody (mAb) were obtained from The Russian Research Center for Molecular Diagnostics and Therapy (Moscow) and are referred to as LS02ABx, LS03ABx, LS04ABx, LS05ABx, LS06ABx, LS07ABx, LS08ABx, LS10ABx, LS11ABX, and LS13ABx. Bovine serum albumin (BSA); goat anti-mouse immunoglobulin G (whole molecule) conjugated to horseradish peroxidase (IgG–peroxidase) #A-4416; polyoxyethylene sorbitan monolaurate (Tween-20); Protein-G conjugated Sepharose #P-32196; 0.02 M TRIS-buffered saline (TBS) #T-5912; 0.9% NaCl, pH 7.4; and 0.01 M phosphate buffered saline (PBS), 0.138 M NaCl, 0.0027 M KCl, pH 7.4 #P-3813; were purchased from Sigma Chemical Co. (St. Louis, MO, USA). HRP conjugated to Streptavidin #SNN2204 was obtained from Invitrogen Inc. (Carlsbad, CA, USA). Black, Maxisorp 96-well Nunc microtiter plates were obtained from PGC Scientific (Gaithersburg, MD, USA), and SuperSignal Pico and Femto Max Sensitivity substrates was purchased from Pierce Inc. (Rockford, IL, USA). Carnation non-fat dry milk (NFDM) was obtained locally. The NuPAGE 4–12% Bis-Tris gels and SilverXpress kit (LC6100) were supplied from Invitrogen. Luminescence was measured using a Perkin-Elmer Victor-3 microplate reader. Data were exported to Microsoft Excel for further analysis.

### 4.2. Monoclonal Antibody Procedure and Purification

Hybridoma cells were grown using our standard hybridoma media in a 4% CO_2_ at 37 °C as previously described [23]. Small amounts (usually less than 10 mL) of ascites fluids were obtained (Covance Research Products, Inc., Denver, PA, USA). Antibodies were purified from the ascites fluid by affinity chromatography on Protein-G Sepharose. Bound antibody was eluted with 0.1 M glycine–HCl, pH 2.7 and then dialyzed against PBS. Protein concentrations were determined with a BCA-kit (Pierce) using the microplate method suggested by the manufacturer. Antibodies were conjugated with biotin using EZ-Link Sulfo-NHS-LC-Biotin (Pierce) as described using a 50-fold molar excess of biotin reagent. Biotin conjugation was evaluated using the Pierce Biotin Quantitation kit according to the manufacturer’s protocol (Pierce, 28005) (based on Beer Lambert Law: **A_λ_** = ε_λ_**bC**). The calculations used were : (1) mmol protein per mL = [protein concentration (mg/mL)/MW of protein (mg/mmol)] = *Calc#1*; (2) ∆A500 = (A500 H\A) − (A500H\A\B) = *Calc#2*; mmol biotin/mL reaction mixture = [∆A500/(34,000 × b)] = *Calc#3*; and mmol biotin in original sample/mmol protein in original sample = [(*Calc#3*) × 10 × dilution factor]/*Calc#1*.

Antibody isotype was determined by ELISA using toxin-coated microtiter plates and horseradish peroxidase-conjugated, isotype-specific anti-bodies (SouthernBiotech, Birmingham, AL, USA) and with IsoStrip mouse monoclonal antibody isotyping kit #11 493 027 001 (Roach Diagnostics, Indianapolis, IN, USA) according to the manufacturers.

### 4.3. ELISA Methods

Direct ELISA’s were as previously described [23] using black microtiter plates coated with 100 µL per well of a 1 µg/mL solution of toxin in 0.05 M sodium carbonate buffer, pH 9.6. Biotin-labeled anti-abrin mAbs at 1 µg/mL were used. SuperSignal Femto Max Sensitivity substrate (Pierce, Rockford, IL, USA) was used according to manufacturer’s instructions. The plates were incubated for 3 min at room temperature and luminescent counts recorded using a Victor-3 microplate reader (PerkinElmer Inc., Waltham, MA, USA).

Capture ELISA’s for routine evaluation of antibody pairs were as described [23]. Black microtiter plates were coated with 50 µL/well of a 2 µg/mL solution of anti-abrin mAb in carbonate buffer overnight at 4 °C. Plates were washed with TBST and remaining reactive sites blocked with 3% NFDM-TBST for 1 h at 37 °C. Plates were then washed and toxin added starting at 300 ng/mL followed by a 2-fold dilution series. Zero toxin wells served as control. Biotin-labeled anti-abrin mAb at 5 µg/mL in 3% NFDM-TBST was added and the plates incubated for 1 h at 37 °C. HRP-conjugated Streptavidin (1:20,000 dilution in TBST) was added and the plates incubated for 1 h at 37 °C. The plates were given a final wash and SuperSignal Pico Extended Duration substrate was added. Luminescence measured at 3 min in a Perkin-Elmer Victor-3 microplate reader. The optimized capture ELISA protocol used 50 µL/well of a 2 µg/mL solution of LS04ABx to coat microtiter plates and biotin labeled detector antibody LS13ABx was used at 1 µg/mL. SuperSignal Femto Max Sensitivity substrate was used and plates were washed 12 times following the streptavidin step in order to lower non-specific signal.

### 4.4. SDS-PAGE Electrophoresis for Silver Stain and Western Blotting

Ricin, abrin, abrin A-chain, abrin B-chain at a concentration of 500 ng per well in the presence or absence of reducing agent (0.05 M DTT) were separated by sodium dodecyl sulfate (SDS)-polyacrylamide gel electrophoresis (PAGE) with NuPAGE 4–12% Bis-Tris gels (Invitrogen) followed by either Silver stain or Western blotting. One gel was subsequently silver stained using the SilverXpress kit according to the manufacturer’s instructions (Invitrogen). For the remaining gels, the resolved proteins were transferred to a PVDF membrane (Invitrogen iBlot). The membranes were blocked in 5% milk-Tris-buffered saline-0.1% Tween 20 buffer then probed with each of the 10 monoclonal LSABx antibodies at a concentration of 5 μg/mL in blocking solution followed by secondary antibody (goat anti-mouse horseradish peroxidase (HRP)-conjugated; 1:3000, Cell Signaling Technology). The blot was incubated in Pierce ECL Western Blotting Substrate solution (Thermo Scientific). Protein bands from peroxidase activities to chemiluminescent substrates were developed and detected using the ChemiDoc MP imaging system (BIO-RAD, Hercules, CA, USA). Molecular weight standards were purchased from Invitrogen.

### 4.5. Capture ELISA with Abrin/Ricin near Neighbor Extracts

To evaluate the specificity of the monoclonal abrin antibodies against other plant ribosome-inactivating proteins (RIPs), extracts from 46 abrin and ricin near neighbors were obtained from Julie R. Avila (Lawrence Livermore National Laboratory, Livermore, CA, USA) as described [24]. Crude extracts were at either 13.2 μg/mL or 20 μg/mL. The most sensitive capture ELISA used SuperSignal Femto Max Sensitivity substrate and plates were washed 12 times following the streptavidin step in order to lower non-specific signal.

### 4.6 Vero Cell Cytotoxicity Assay

Vero cells were cultured in Dulbecco’s Modified Eagle Medium (DMEM) high glucose +10% fetal bovine serum and incubated in a humidified incubator (37 °C, 5% CO_2_). Cells were trypsinized and adjusted to 0.5 × 10^5^ cells/mL, seeded into black-sided, clear-bottom 96-well tissue culture plates at 100 µL/well and allowed to adhered overnight (18 h) at 37 °C. The media was then removed, and 100 µL of fresh DMEM containing abrin or ricin with and without mAb dilutions (pre-incubated 1 h at 37 °C) was then added. Plates were then incubated at 37 °C for 2 h, the media was removed and fresh media added. The cells were then incubated for 48 h at 37 °C. Cytotoxicity was measured as follows: 100 µL of CellTiter-Glo (Promega, Madison, WI, USA, G7570) at 1:5 in PBS was added to each well and the plates shaken for 2 min to lyse the cells. After 10 min at room temperature, luminescence was measured using a Victor-3 plate reader (lid was removed from the plate for a better signal). Statistical significance was calculated using the two-tailed unpaired Student’s *t*-test with *p*-values < 0.05 considered significant.

## Figures and Tables

**Table 1:** Isotypes and biotin loading of anti-abrin monoclonal antibodies.

Monoclonals	Isotype	Biotin/IgG
LS02ABx	IgG1, kappa	4.0 ± 0.17
LS03ABx	IgG1, kappa	3.9 ± 0.10
LS04ABx	IgG2a, kappa	3.8 ± 0.20
LS05ABx	IgG2b, kappa	2.5 ± 0.50
LS06ABx	IgG1, kappa	1.5 ± 0.5
LS07ABx	IgG1, kappa	3.7 ± 0.3
LS08ABx	IgG2a, lambda	4.0 ± 0.16
LS10ABx	IgG1, kappa	2.5 ± 0.5
LS11ABx	IgG1, kappa	2.0 ± 0.01
LS13ABx	IgG1, kappa	1.8 ± 0.20

**Table 2:** Observed LODs (ng/mL) for all possible combinations of anti-Abrin mAbs.

Detectorm Abs										
	LS02ABx	LS03ABx	LS04ABx	LS05ABx	LS06ABx	LS07ABx	LS08ABx	LS10ABx	LS11ABx	LS13ABx
Capture mAbs										
LS02ABx	-	200	200	-	-	-	50	-	-	-
LS03ABx	100	-	-	-	200	200	-	-	-	200
LS04ABx	-	-	-	200	-	-	-	-	5	10
LS05ABx	-	-	-	-	-	-	-	-	-	-
LS06ABx	-	-	-	-	-	75	10	100	-	-
LS07ABx	-	-	-	-	-	75	40	-	-	-
LS08ABx	100	40	-	100	-	100	-	75	5	50
LS10ABx	-	-	-	-	-	-	-	-	-	-
LS11ABx	-	-	-	-	-	-	30	-	-	-
LS13ABx	100	200	200	-	-	-	100	-	-	-

The minus sign indicates limits of detection (LODs) greater than 300 ng/mL.

**Table 3:** Results of Abrin capture ELISA with near neighbor extracts.

Near Neighbours	Capture ELISA
*Abrus laevigatus* E. Mey.	-
*Abrus precatorius* L. +	+
*Abrus schimperi* subsp*. Africanus* (Vatke) Verdc.	-
*Acalypha rhomboidea* Raf.	-
*Acalypha rhomboidea* 11837	-
*Adriana quadripartite* (Labill.) Gaudich.	-
*Adriana quadripartita* 76851	-
*Bryonia dioica* Jacq.	-
*Canavalia gladiate* (Jacq.) DC.	-
*Canavalia rosea* (Sw.) DC	-
*Canavalia madagascariensis* J.D. Sauer	-
*Cinnamomum camphora* (L.) J.Presl.	-
*Cucurbita moschata* Duchesne	-
*Dianthus caryophyllus* Linnaeus	-
*Fatsia japonica* (Thunb.) Decne & Planch.	-
*Fatsia japonica* 39610	-
*Galactia striata* (Jacq.) Urban	-
*Galactia wrightii* A. Gray	-
*Iris hollandica* Bf.	-
*Jubernardia globifera* (Benth.) Troupin	-
*Jubernardia globifera* 76851	-
*Luffa acutangula* (L.) Roxb.	-
*Luffa cylindrical (aegyptica)* Bergquist, 1995	-
*Lychnis chalcedonica* (L.) E.H.L. Krause	-
*Macaranga grandifolia* (Blanco) Merr.	-
*Macaranga grandifolia* 3403	-
*Mallotus nudiflorus* (L.) Kulju & Welzen	-
*Mallotus philippensis* (Lam.) Müll. Arg.	-
*Manihot escuelenta* Crantz.	-
*Manihot escuelenta* 13562	-
*Mercurialis annua* L.	-
*Mercurialis annua* 279720	-
*Momordica charantia* L.	-
*Phytolacca Americana* L.	-
*Phytolacca americana* 39161	-
*Phytolacca dioica* L.	-
*Plukenetia volubilis* 72130	-
*Plukenetia volubulis* L.	-
*Sambucus ebulus* L.	-
*Sambucus nigra* L.	-
*Saponaria officinalis L.*	-
*Saponaria officinalis* 391915	-
*Senna occidentalis* (L.) Link	-
*Trewia nudiflora* 85123	-
*Trichosanthes kirilowi* Maxim.	-
*Viscum album* L.	-
*Viscum album* 22397	-

**-** = Zero activity at 3 μg/mL of sample in capture ELISA. + = Activity at 3 μg/mL of sample in capture ELISA.

**Table 4:** Neutralization of abrin cytotoxicity with monoclonal antibodies. Values represent means of two samples ± SD. Statistical significance was determined by two-tailed unpaired Student’s *t*-test, *p* < 0.05 for all conditions compared to no toxin (DMEM) except for LS07ABx, LS02ABx, and LS02 + 07 + 13.

Treatment	Relative Toxicity (%)
DMEM	0
Abrin	100
LS07ABx	4 + 3
LS13ABx	4 ± 1
LS02ABx	4 ± 3
LS02 + 07 + 13	4 ± 3
LS04Abx + Abrin	94 ± 1
LS07Abx + Abrin	69 ± 2
LS08Abx + Abrin	88 ± 1
LS10Abx + Abrin	94 ± 1
LS13Abx + Abrin	54 ± 1
LS11Abx + Abrin	77 ± 1
LS03Abx + Abrin	96 ± 0
LS02Ax + Abrin	66 ± 2
LS02 + 07 + 13 + Abrin	41 ± 2

## Supplementary Materials

The following are available online at www.mdpi.com/2072-6651/9/10/328/s1, Table S1: Ricin cytotoxicity is not neutralized with LSABx monoclonal antibodies; Supplemental Figure S1: Two independent experiments for capture ELISA.
