# Supplementary material for: A Monoclonal–Monoclonal Antibody Based Capture ELISA for Abrin
Source: Toxins (Basel). 2017 Oct 18;9(10):328. doi: 10.3390/toxins9100328 (PMC5666375; doi:10.3390/toxins9100328)
Supplement: Supplementary file 1 [file toxins-09-00328-s001.pdf]

Supplementary Materials: A Monoclonal–Monoclonal Antibody Based Capture ELISA for Abrin

Christina C. Tam, Luisa W. Cheng , Xiaohua He , Paul Merrill , David Hodge and Larry H. Stanker

Table S1: Ricin cytotoxicity is not neutralized by LSABx mAbs. In these experiments, 50 μg/mL of monoclonal antibodies were mixed with and without 5 ng/mL of ricin. Treatment with ricin alone was set as 100% relative toxicity. Values represent means of six samples ± SD. Statistical significance was determined by two-tailed unpaired Student's *t*-test, $p < 0.0001$ for all conditions compared with ricin alone.

Treatment	Relative Toxicity (%)
DMEM	0
Ricin	100
LS02ABx	0
LS07ABx	0
LS13ABx	0
LS02+07+13	0
LS13ABx+Ricin	97 ± 0.46
LS02+07+13+Ricin	100 ± 0.44

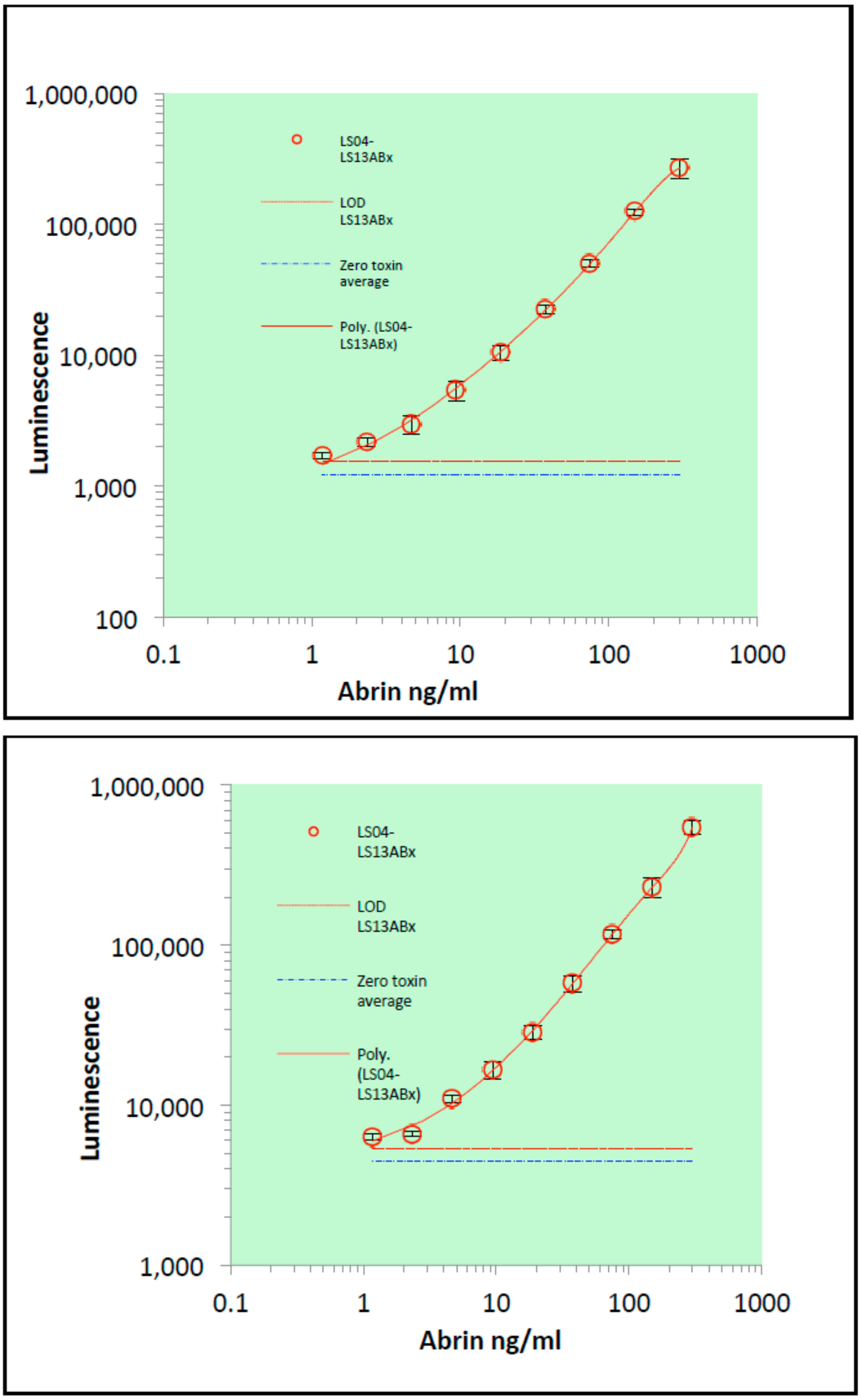

Figure S1: Two independent experiments for capture ELISA. Mab LS04ABx used as the capture reagent at 2 µg/mL. Detector antibody LS13ABx was used at 1 µg/mL. Graphs represent data from two independent experiments performed on separate days with different toxin stocks and buffer. Points represent the average of the replicates (n=6), error bars = standard deviation. Solid lines show 4-Parameter curve fitting. Red dashed line represents the lowest detection level cut offs calculated as the average of the zero plus three standard deviations. Blue broken line represents the average value of the zero toxin control. Statistical significance was determined by two-tailed unpaired Student's *t*-test, $p < 0.05$ considered statistically significant for all points compared to the average zero toxin value.
